# A New Glycosyltransferase Enzyme from Family 91, UGT91P3, Is Responsible for the Final Glucosylation Step of Crocins in Saffron (*Crocus sativus* L.)

**DOI:** 10.3390/ijms22168815

**Published:** 2021-08-16

**Authors:** Alberto José López-jimenez, Sarah Frusciante, Enrique Niza, Oussama Ahrazem, Ángela Rubio-Moraga, Gianfranco Diretto, Lourdes Gómez-Gómez

**Affiliations:** 1 Instituto Botánico, Departamento de Ciencia y Tecnología Agroforestal y Genética, Universidad de Castilla-La Mancha, Campus Universitario s/n, 02071 Albacete, Spain; albertojose.lopez@uclm.es (A.J.L.-j.); enrique.niza@uclm.es (E.N.); oussama.aharazem@uclm.es (O.A.); angela.rubio@uclm.es (Á.R.-M.); 2 Casaccia Research Centre, Italian National Agency for New Technologies, Energy, and Sustainable Development, 00123 Rome, Italy; sarah.frusciante@enea.it (S.F.); gianfranco.diretto@enea.it (G.D.); 3 Escuela Técnica Superior de Ingenieros Agrónomos y de Montes, Campus Universitario s/n, 02071 Albacete, Spain; 4 Facultad de Farmacia, Campus Universitario s/n, 02071 Albacete, Spain

**Keywords:** apocarotenoids, carotenoids, Crocus, glucosyltransferases, saffron, stigma

## Abstract

Crocetin is an apocarotenoid formed from the oxidative cleavage of zeaxanthin, by the carotenoid cleavage enzymes CCD2 (in Crocus species) and specific CCD4 enzymes in Buddleja davidii and Gardenia jasminoides. Crocetin accumulates in the stigma of saffron in the form of glucosides and crocins, which contain one to five glucose molecules. Crocetin glycosylation was hypothesized to involve at least two enzymes from superfamily 1 UDP-sugar dependent glycosyltransferases. One of them, UGT74AD1, produces crocins with one and two glucose molecules, which are substrates for a second UGT, which could belong to the UGT79, 91, or 94 families. An in silico search of Crocus transcriptomes revealed six candidate UGT genes from family 91. The transcript profiles of one of them, UGT91P3, matched the metabolite profile of crocin accumulation, and were co-expressed with UGT74AD1. In addition, both UGTs interact in a two-hybrid assay. Recombinant UGT91P3 produced mostly crocins with four and five glucose molecules in vitro, and in a combined transient expression assay with CCD2 and UGT74AD1 enzymes in Nicotiana benthamiana. These results suggest a role of UGT91P3 in the biosynthesis of highly glucosylated crocins in saffron, and that it represents the last missing gene in crocins biosynthesis.

## 1. Introduction

**Figure 1:**
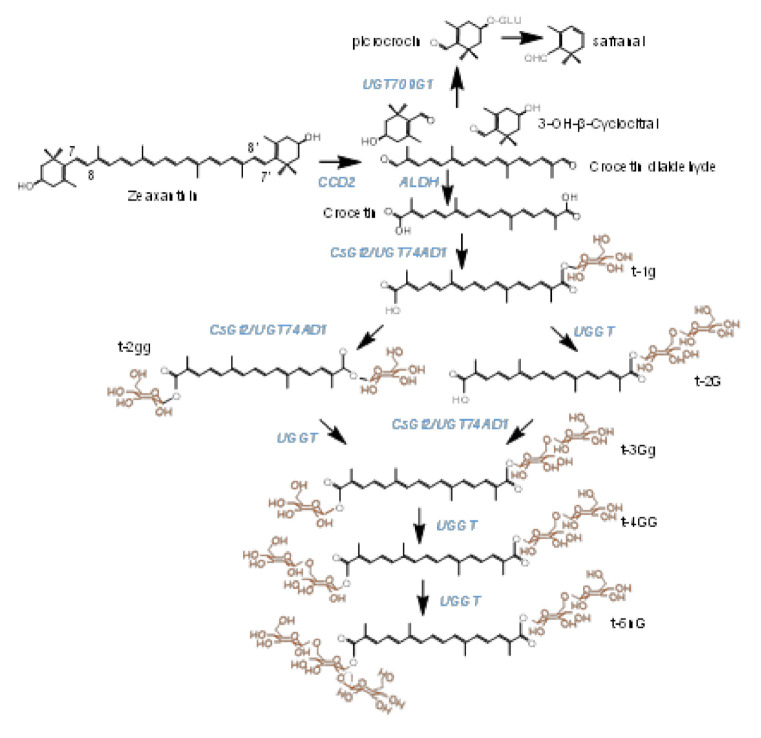
Biosynthetic pathway of crocins in saffron stigmas, from the zeaxanthin precursor. Enzymes catalyzing each step in the pathway are highlighted in blue. The target enzymes in this study are named UGGT. Glucose molecules conferring solubility in water to crocins are highlighted in orange.

Crocins (crocetin esters) are glycosylated apocarotenoid compounds present in a limited group of plants [1]. The presence of these pigments in flowers and fruits suggests their implications in pollinator- and seed-disperser attraction [2]. Crocins accumulate at huge levels in the stigmas of saffron (*Crocus sativus* L.), and are responsible for the bright red color showed by the saffron spice, one of the most appreciated in the world. These hydrophilic apocarotenoids have been extensively used by food and pharmaceutical industries, due to their colorant and medicinal properties, respectively [3]. Crocins with four (crocetin-di-(β-D-gentiobiosyl)-ester) and three (crocetin-(β-D-gentiobiosyl)-(β-D-glucosyl)-ester) glucose residues are the main crocins present in saffron (Figure 1) [4,5]. Crocetin, the direct precursor of crocins, is generated in the frame of the carotenoid catabolic pathway, whose flux is controlled in saffron by four phytoene synthase genes (PSY1a, PSY1b, PSY2 and PSY3), characterized by different expression patterns [6]. In more detail, crocetin results from the cleavage of zeaxanthin, in a reaction catalyzed by carotenoid cleavage dioxygenase enzymes (CCDs). There are four main CCD subfamilies in plants (CCD1, CCD4, CCD7 and CCD8), which catalyze different cleavage reactions over several carotenoid and apocarotenoid compounds [7]. In saffron and other Crocus species, the enzyme catalyzing crocetin biosynthesis is closely related to the CCD1 subfamily, and named CCD2 [8,9], while a different CCD, related to the CCD4 subfamily, is responsible for crocetin biosynthesis in the medicinal plant *Buddleja davidii* [1]. This cleavage step of zeaxanthin at the 7,8;7′,8′ double bonds takes place in the chromoplast [9], and originates crocetin dialdehyde and 2,6,6-trimethyl-4-hydroxy-1-carboxaldehyde-1-cyclohexene (HTCC). Subsequently, crocetin dialdehyde is converted to crocetin by a reaction catalyzed by ALDHs [10], and further, is successively glycosylated by the addition of glucose moieties to yield crocins, which are stored in the vacuole [11,12]. More than 20 years ago, Dufresne and col. [13] proposed the presence in saffron of two crocetin glucosyltransferases activities, UDP-glucose:crocetin 8,8′-O-glucosyltransferase and UDP-glucose: crocetinglucosylester 6″-O-glucosyltransferase, leading to the formation of crocins with different glucosylation degrees. The UDP-glucose:crocetin 8,8′-O-glucosyltransferase has been identified in saffron [4] and in other Crocus species [14], but whether the second proposed enzyme is present is still unknown. Another plant known to accumulate crocins is *Gardenia jasminoides* [15], and their formation is catalyzed by four different UGTs, UGT75L6 and UGT94E5 [16], and GjUGT74F8 and GjUGT94E13 [17]. The expression patterns of neither UGT94E5 nor UGT75L6 correlated with the pattern of crocin accumulation in the fruits of *G. jasminoides* [16], and the expression levels in this organ are low. By contrast, GjUGT74F8 and GjUGT94E13 are highly expressed in green and red fruits [17]. More recently, three UGTs involved in the biosynthesis of crocins have been identified in *Buddleja davidii*, BdUGT74BC1, BdUGT74BC2 and BdUGT94AA3 [18], and their expression patterns are correlated with the accumulation of crocins in the flowers of Buddleja. As for the case of GjUGT94E13, the enzyme BdUGT94AA3 catalyzes the glucosylation of crocins with one, two and three glucose molecules.

The glycosylation of small molecules, which have effects on their stability, localization, chemical properties, and bioactivity, is one of the most prevalent modifications in plant secondary metabolism [19]. This reaction is catalyzed by UDP-glucose-dependent glycosyltransferases (UGTs), which are widespread in plants with many different roles [20,21]. The characteristic feature of these UGTs is the conserved C-terminal plant secondary product glycosyltransferase (PSPG) motif, important for interactions with nucleotide diphosphate sugar. UGTs comprise the largest family (GT family 1) inside the glycosyltransferase superfamily, and are encoded by a large number of genes in the sequenced plant genomes (https://phytozome.jgi.doe.gov/, accessed on 1 January 2021). This family exhibits inverting mechanism of catalysis and depict a GT-B structural fold. UDP-glucose is the primary sugar donor in family 1, followed by UDP-galactose, UDP-rhamnose, UDP-xylose and UDP-glucuronic acid [22]. Members of the family 1 UGTs display high variability in their substrate specificity, represented by a wide range of plant secondary metabolites such as alkaloids, betacyanin, cyanohydrins, phenolics, sterols, terpenes, and thiohydroximates, despite the conserved secondary and tertiary structures of these enzyme [23]. Some UGTs glycosylate a broad range of acceptor molecules, while others act only on one or a few substrates [23]. The substrate promiscuity of UGTs make it difficult to predict the biologically relevant in vivo functions of UGTs based on primary sequence data alone, and functional analysis is required [24]. Further, UGTs not only act as monosaccharide enzymes, but also function as disaccharide or trisaccharide glycoside-forming enzymes. Characteristic sugar–sugar UGTs mainly belong to the UGT94, UGT73, UGT79, and UGT91 families [25]. These enzymes could add a glucosyl, glucuronosyl, xylose, rhamnosyl, or galactosyl molecule to an existing sugar moiety in different metabolites. Several features at the amino acid level are observed among these enzymes, while the monoglycoside UGTs contain a special feature at the C-terminal end, consisting of the GSS motif, and the disaccharide UGTs enzymes, in general, contain highly conserved amino acids, such as a methionine at position 25, a conserved leucine at position 29, and proline at position 90 [26].

Crocins biosynthesis is developmentally regulated in the stigma of saffron [27]. With the production of large transcriptomic datasets, co-expression analysis is becoming a powerful approach for the identification of candidates for several metabolic pathways in plants [28]. In this study, we took advantage of a large saffron transcriptome dataset to identify candidate crocin UGTs. In a previous work on crocin biosynthesis, we characterized UGT74AD1, which allows the formation of crocins with one and two glucose molecules [4]. Here, we describe the discovery and characterization of the next candidate UGT, named UGT91P3, which unable to use crocetin as a substrate, but can produce crocins with more than two glucose molecules. The newly discovered enzyme was biochemically and functionally characterized, and its ability for crocin glucosylation was demonstrated in vitro and in *Nicotiana benthamiana* plant experiments. Overall, our data confirm that UGT91P3 is a key enzyme in crocin biosynthesis in saffron, it being the last enzymatic step of these high-value apocarotenoids, and providing a new tool for the development of these soluble apocarotenoids in diverse heterologous systems.

## 2. Results

### 2.1. Dentification of Candidate Glycosyltransferases by Expression Analyses, Crocins Accumulation and Co-Expression with UGT74AD1

To identify candidate genes involved in the glucosylation of crocins, we used a large stigma RNA-seq transcriptome dataset from saffron [29,30]. The as-yet identified glycosyltransferases that catalyze the glycosylation of the sugar moiety attached to aglycones belong to UGT73, UGT79, UGT91, and UGT94 families. Therefore, we focused on genes with similarity to UGTs in the above families as potential candidate genes. Six nucleotide sequences encoding for UGTs that belong to the UGT91 subfamily were identified as possible candidates for crocin glucosylation in the stigmas of saffron (c367693_g2_i1, c411064_g1_i1, c411064_g2_i1, c370708_g1_i2, c370708_g1_i1, c365848_g1_i1) (Supplementary Figure S1), while no sequences were identified with similarities to UGT73, UGT79 and UGT94. The selected sequences were 1392–1413 base pairs long-encoding proteins of 464–471 amino acids. Pairwise amino acid sequence analyses showed 93.06% identity between c367693_g2_i1 and c411064_g2_i1; 96.92% identity between c411064_g1_i1 and c365848_g1_i1, and 90.46% identity between c370708_g1_i2 and c370708_g1_i1 (Supplementary Figure S1). The consensus PSPG box sequence of these enzymes from saffron is shown in Supplementary Figure S1C and can be compared with the PSPG box consensus sequence obtained for UGTs from subfamilies 79, 91 and 94 (Supplementary Figure S2). The amino acid residues in positions 18 and 31 were conserved between the UGTs from saffron and UGT91 (Supplementary Figures S1 and S2). Interestingly, the amino acid residue in position 22 was not conserved among the saffron UGTs, as observed for the subfamily UGT79, in contrast with the UGT94 and UGT91 subfamilies (Supplementary Figures S1 and S2). The names for these enzymes were designated as UGT91P3 (GenBank accession number: MZ190170) for c367693_g2_i1, UGT91P4 (GenBank accession number: MZ190171) for c411064_g1_i1, UGT91P5 (GenBank accession number: MZ190172) for c411064_g2_i1, UGT91P6 (GenBank accession number: MZ190175) for c365848_g1_i1, UGT91K2 (GenBank accession number: MZ190173) for c370708_g1_i1, and UGT91K3 (GenBank accession number: MZ190174) for c370708_g1_i2; according to the UDP Glucuronosyltransferase Nomenclature Committee (http://www.cazy.org/GlycosylTransferases.html, accessed on 1 December 2021).

The three-dimensional structures of these six enzymes, obtained by homology modelling using the Phyre Sever, were analyzed, and all had the GT-B fold, one of the two main folds for the GT superfamily [31], consisting of two N- and C-terminal domains with Rossmann-like folds that are very similar to each other (Supplementary Figure S3). The N-terminal domain is involved in acceptor sugar molecule binding, and is more variable, consistent with the diversity of acceptor molecules. The C-terminal domain involves the nucleotide diphosphate sugar, and is more conserved than the N-terminal domain, which is consistent with the conserved nature of the sugars. The structures of the six UGTs were also superimposed based on percentage identity to determine any structural characteristic motifs of each UGT (Supplementary Figure S4), and then compared with UGT74AD1 (Supplementary Figure S3). In the calculated 3D structure of UGT74AD1, the two backbone helices of the C-terminal domain (shown in orange and red) were separated by a well-defined loop structure, whereas the other UGTs showed heterogeneous geometries in the linker region of both helices (Supplementary Figure S3), as observed for other di-glucosyltransferases [26]. These proteins are also characterized by an expanded pocket size in the active site of the glycosylation reaction, which accommodates the enlarged glycosylated substrates [32].

**Figure 2:**
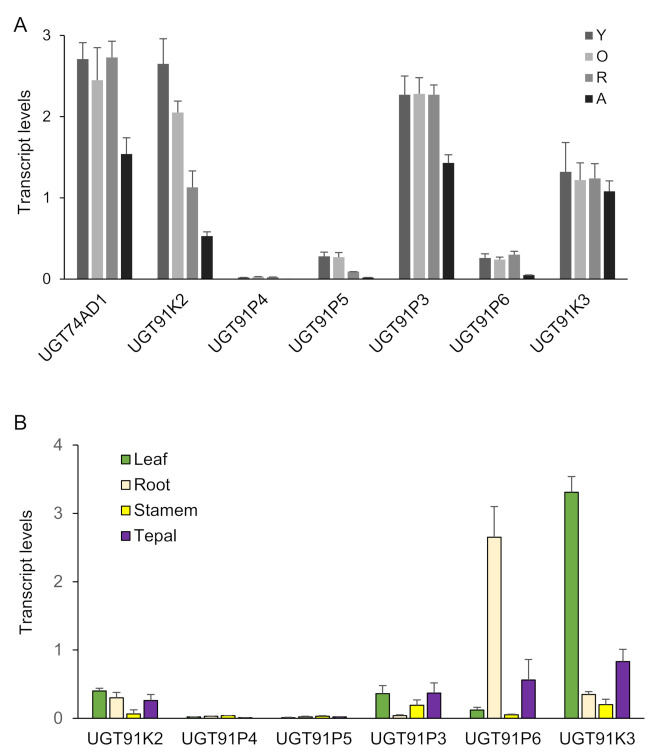
Transcript abundance of saffron genes encoding for putative UGTs. Transcript abundance was determined by qRT-PCR. (**A**) Expression levels in stigma tissue. RNA was isolated from stigmas at four different developmental stages: A (yellow stigma), O (orange stigma), R (red undeveloped stigma), A (stigma at anthesis). (**B**) Expression levels in different vegetative tissues. Means and standard errors are shown (*n* = 3).

Next, we analyzed the expression of these six genes in four developmental stages of saffron stigmas (Figure 2A), and compared them with the expression levels in these stages of CsGt2 (UGT74AD1), considering that enzymes catalyzing sequential reactions may be co-expressed. Three genes, UGT91K3, UGT91K2 and UGT91P3, showed expression levels similar to UGT74AD1, and their expressions were developmentally regulated, with higher expression levels in the initial developmental stages (yellow, Y; orange, O; and red, R) (Figure 2A). In addition, the transcript levels of the candidate genes were analyzed in different vegetative tissues (Figure 2B). The UGT91K3 and UGT91P6 genes showed higher levels of expression in leaves and roots compared with the stigma, respectively (Figure 2A,B). Therefore, they did not appear to have a preferential site of expression in the crocins-accumulating tissue. On the contrary, UGT91K2 and UGT91P3, which showed high expression levels in the stigma tissue, were characterized by low expression levels in all the vegetative tissues analyzed (Figure 2B).

**Figure 3:**
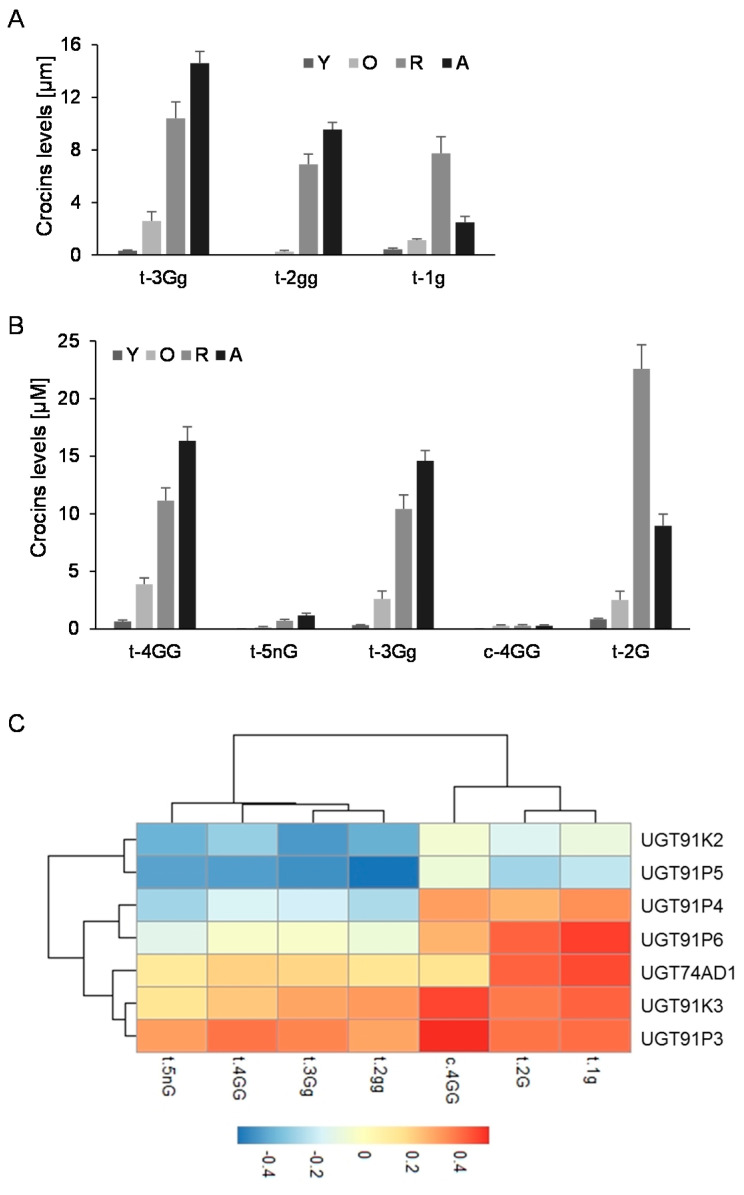
Levels of crocins in stigma tissue and the correlation with the expression levels of saffron UGTs. (**A**) Accumulation of crocins possibly acting as substrates for UGT enzymes in saffron stigmas at different developmental stages. (**B**) Accumulation of crocins with more than one glucose molecule at their ends, which could be the products of the reactions catalyzed by the UGT enzymes in saffron stigmas at different developmental stages. (**C**) Transcript and crocins data were integrated using the Pearson correlation coefficients (ρs), and the obtained data were plotted to visualize positive and negative correlations using pheatmap Package version 1.0.12. (https://CRAN.R-project.org/package=pheatmap, accessed on 20 June 2021).

Further, we analyzed the levels of crocins that could act as substrates for the UGTs encoded by these genes (1g (crocetin-(β-D-glucosyl)-ester); 2gg, crocetin-di-(β-D-glucosyl)-ester; 3Gg (crocetin-(β-D-gentiobiosyl)-(β-D-glucosyl)-ester)) (Figure 3A), and the possible resulting crocins after their glucosylation (Figure 3B): (2G, crocetin-(β-D-gentiobiosyl)-ester); 3Gg (crocetin-(β-D-gentiobiosyl)-(β-D-glucosyl)-ester); 4GG (crocetin-di-(β-D-gentiobiosyl)-ester; and 5nG (crocetin-(β-D-gentiobiosyl)-(β-D-neapolitanosyl)-ester). We performed a co-expression correlation analysis, by integrating the transcript levels of each gene into the data for the different developmental stages of the stigma and crocins levels, and calculating, for each data pair, the corresponding Pearson correlation coefficient (Figure 3C). Four out of the six genes showed positive correlations with crocin evolution in the stigma tissue, UGT91P3, UGT91P4, UGT91K3, and UGT91P6; interestingly, of these four, only two genes, UGT91P3 and UGT91K3, displayed positive correlations with all the crocins under study, and thus might act as substrates for these enzymes (Figure 3C). Based on these results, together with the expression levels in the stigmas and in vegetative tissues (the latter being tissues not accumulating crocins [29]), we decided to focus our attention on UGT91P3, by carrying out in bacteria and in planta functional characterization.

### 2.2. UGT91P3 Catalyzes the Formation of Crocins with Three, Four and Five Glucose Molecules

The full-length open reading frame for UGT91P3 encodes a putative protein of 464 amino acids and 50.8 kDa. A BLAST search (https://blast.ncbi.nlm.nih.gov/Blast.cgi, accessed on 10 July 2021) revealed the 55% identity of UGT91P3 with a predicted UGT from Phoenix dactylifera (XP_008788932.1). Additionally, UGT91P3 did not show any signal peptide, as predicted by TargetP (http://www.cbs.dtu.dk/services/TargetP/, accessed on 10 July 2021).

**Figure 4:**
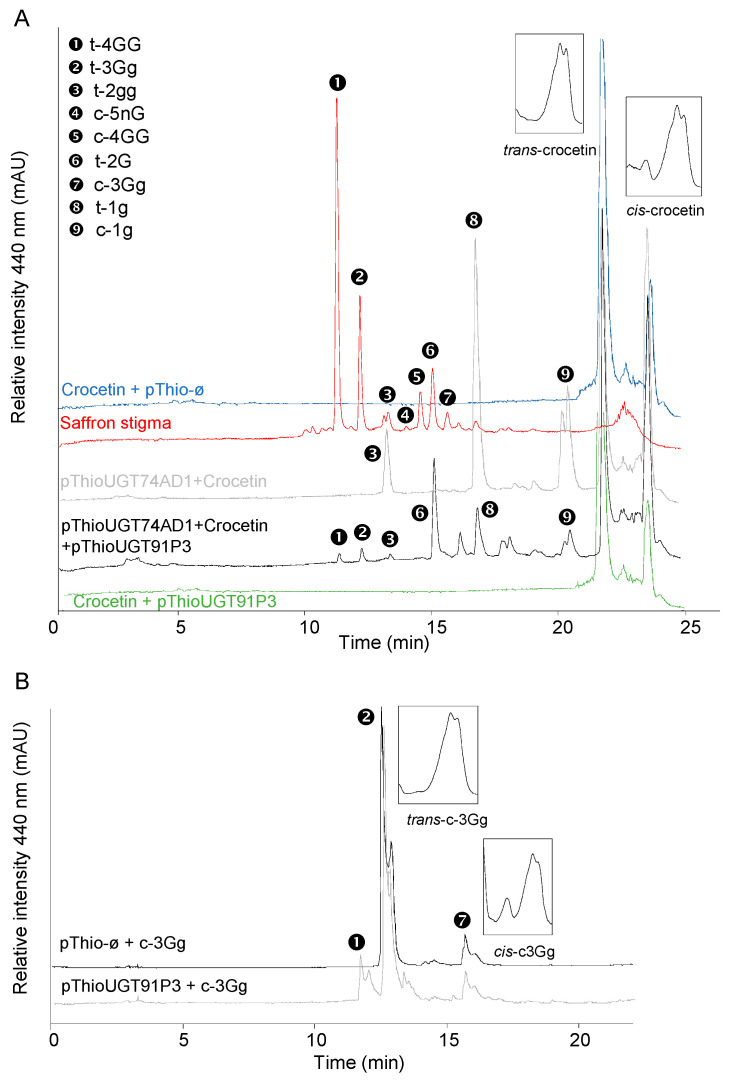
Enzyme activity of UGT91P3. The UGTs were expressed in *E. coli* and protein extracts were assayed for activity with crocetin and crocins. (**A**) Enzyme assays using crocetin as substrate. (**B**) Enzyme assays using crocin t-3Gg as substrate. As controls, all the assays were performed with protein extracts from *E. coli* transformed with an empty vector. Products were analyzed using HPLC-DAD and HESI-HRMS. UV detection was monitored at 440 nm.

To test UGT91P3’s putative activity for crocin glucosylation, the full-length cDNA was amplified using RNA from stigma tissue in the orange stage. Upon cloning and sequencing the obtained amplicon, the corresponding clone was used for recombinant protein expression and enzyme characterization in *Escherichia coli*. To this end, total protein extracts were used for enzyme activity with crocetin as the substrate, followed by HPLC-DAD analyses, but no products were detected (Figure 4A). Next, we carried out the assay of UGT91P3 over crocetin, but adding to the reaction the enzyme UGT74AD1. This enzyme catalyzed the production of crocins with one (1g (crocetin-(β-D-glucosyl)-ester)) and two glucose molecules (2gg, crocetin-di-(β-D-glucosyl)-ester) from crocetin (Figure 4A and Table 1); HPLC-DAD analyses of the products obtained revealed the presence of new products, identified by LC-DAD-HESI-HRMS analyses (Figure 4A and Table 1), corresponding to trans-crocins with four glucose molecules (crocetin-di-(β-D-gentiobiosyl)-ester), three glucosde molecules, crocetin-(β-D-gentiobiosyl)-(β-D-glucosyl)-esters; and with a gentiobiose molecule (crocetin-(β-D-gentiobiosyl)-ester) as the major products. In addition, and most notably, the LC-DAD-HESI-HRMS analyses of the products revealed the presence of crocetin with five glucose molecules (crocetin-(β-D-gentiobiosyl)-(β-D-neapolitanosyl)-ester). The activity of UGT91P3, without UGT74AD1, was further tested over crocin 3Gg (crocetin-(β-D-gentiobiosyl)-(β-D-glucosyl)-ester)), followed by HPLC-DAD analyses of the products and further HESI-HRMS analyses, showing the formation of crocetin-di-(β-D-gentiobiosyl)-ester (Figure 4B and Table 1) and crocetin-(β-D-gentiobiosyl)-(β-D-neapolitanosyl)-ester, as detected in the HESI-HRMS analyses (Table 1). Crocetin and crocin 3Gg were also incubated under the same conditions with empty vector (control) protein extracts (Figure 4A,B), and no products were produced in these control samples.

### 2.3. Three Alleles of UGT91P3 Are Expressed in Stigmas and Showed Differential Activity

The amplified band obtained from orange stigmas was used for the isolation of additional UGT91P3 alleles, due to the triploid nature of saffron. The band was cloned, and a total of 100 independent colonies were selected and sequenced. The obtained sequences were analyzed and grouped in three different nucleotide sequences in proportions of 1:2:10 (Supplemental Figure S5). The three different sequences showed identities ranging from 95.91 to 98.49% (Supplemental Figure S6). Major differences at the nucleotide and amino acid levels were present between amino acids 156–169 (Supplemental Figure S6), localized in a loop region, named loop N5a (Supplemental Figure S7), which participates in the formation of the sugar acceptor pocket [23]; more specifically, in positions 157 (L changed for F), 159 (S changed for K), 161 (G changed for K/S), and 166 (G changed for D). Larger amino acid residues were found in the newly identified alleles, which could result in restricting the space availability for the binding of the sugar acceptor molecule (Supplemental Figure S7). In addition, the changes were also associated with alterations in hydrophobicity behavior (in position 161 (G changed for K/S)), which might have an effect on correct interaction with the substrate. In order to determine whether these changes affect the activity of these new alleles, they were cloned in pTHIO and tested against crocin 3Gg. None of the new alleles catalyzed the transfer of glucose on crocin 3Gg in the in vitro reactions (Supplemental Figure S8).

### 2.4. Interaction between UGT91P3 and UGT74AD1 Revealed by Yeast Two-Hybrid (Y2H) Analysis

**Figure 5:**
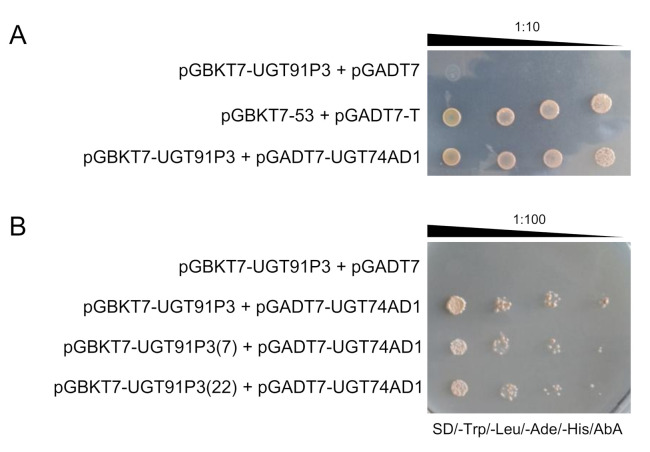
Interaction between UGT91P3 and UGT74AD1 in yeast by two-hybrid (Y2H) analyses. (**A**) Serial dilutions of diploid yeast cells containing different constructs were spotted on selective media, and incubated for two days at 30 °C. The image is representative of four independent experiments, each with 3 different clones analyzed. (**B**) Serial dilutions of diploid yeast cells containing UGT91P3 and the other two alleles (7 and 22) were spotted on selective media, and incubated for two days at 30 °C. The image is representative of four independent experiments, each with 3 different clones analyzed.

As UGT74AD1 and UGT91P3 participate in the same pathway of crocin biosynthesis in saffron (Figure 1), we hypothesized that they could form an enzyme complex to modulate crocins glucosylation. To test this hypothesis, we used yeast two-hybrid (Y2H) analysis to detect protein–protein interactions between UGT74AD1 and UGT91P3. Yeast growth was observed when diploid cells containing both plasmids were placed on selective medium, and their growth was comparable to that of the positive control (pGBKT7-53 and pGADT7-T) (Figure 5), thus suggesting that UGT74AD1 directly interacts with UGT91P3. In addition, the identified alleles for UGT91P3 were also able to interact with UGT94AD1, suggesting that the observed changes in their sequence do not compromise the interaction with UGT74AD1.

### 2.5. In Planta Assessment of UGT91P3 and UGT74AD1 Activities

**Figure 6:**
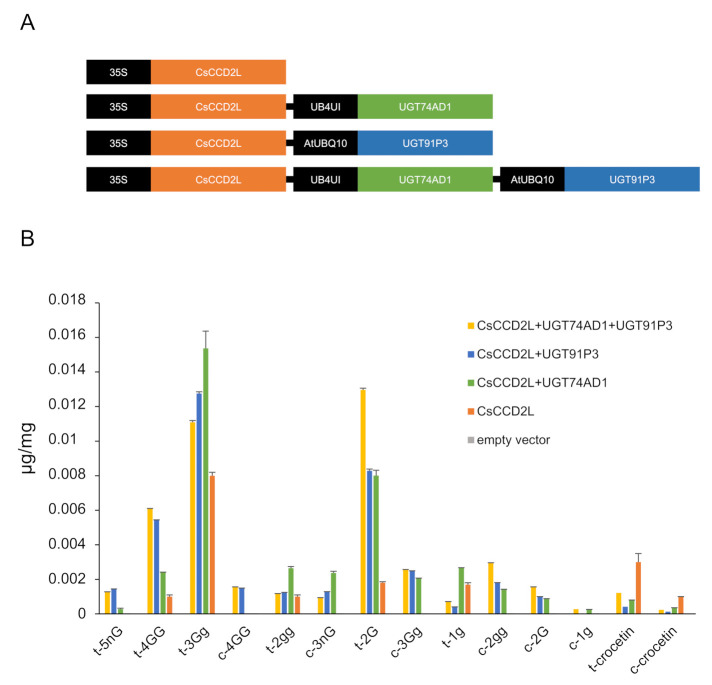
*N. benthamiana* leaves transiently expressing different constructs for crocin production. (**A**) Constructs used for transient expression analyses. (**B**) Tobacco leaves were infiltrated with Agrobacterium transformed with different constructs. Leaves were collected 8 days after infiltration. Metabolites were extracted with methanol:water (50:50), analyzed by LC-HESI-HRMS, and identified based on standard co-elution and their fragmentation patterns. Data are represented as avg std. dev. of at least 3 biological replicates.

To validate the function of UGT91P3 in planta, we transiently transformed 4-week-old *N. benthamiana* plants with plasmids harboring UGT91P3 in different combinations with CsCCD2L [9] and UGT74AD1 [4] (Figure 6A), and evaluated the accumulation of crocins in the agro-infiltrated tissues (Figure 6B). We analyzed polar extracts of leaves harvested 8 d after agroinfiltration by LC-DAD-HESI-HRMS, which displayed the production, at a higher accumulation level, of crocins with four and five glucose molecules, as well as an increased content of crocin 2G. The lower levels of crocin 3 Gg in favor of 4 GG and 5 nG in leaves agroinfiltrated with UGT91P3 suggest that the former could act as the preferred substrate for this enzyme for the production of crocins with a higher glucosylation degree (Figure 6B).

## 3. Discussion

Crocins are characteristically non-volatile glucosylated apocarotenoids that confer the red color of saffron stigmas. The glycosylation of secondary metabolites is usually catalyzed by uridine diphosphate-dependent glycosyltransferases (UGTs) that belong to the carbohydrate-active enzyme (CAZY) glycosyltransferase 1 (GT1) family [20]. Here, we report the identification of a novel enzyme, named UGT91P3 according to the UGT nomenclature, which, together with the other previously characterized crocin pathway genes, is preferentially expressed in the stigma tissue, the site of accumulation of these apocarotenoids in saffron [5]. We exploited this highly tissue-specific expression pattern to identify potential UGT candidate genes using a transcriptome mining approach [33]. The search was focused on transcripts encoding for putative UGTs from UGT94, UGT73, UGT79, and UGT91 subfamilies. A total of 6 transcripts, predicted to encode full-length sequences for UGTs, were obtained, and all were shown to belong to the UGT91 subfamily. Enzymes catalyzing sequential reactions in the biosynthesis of plant-specialized metabolites, such as crocins, may be co-expressed, and such co-expression can be explored for gene discovery. For these reasons, the expression patterns of the identified genes were analyzed in several tissues, including the stigma at different developmental stages, revealing that the genes for 3 of them (*UGT91K2*, *UGT91K3* and *UGT91P3*) had expression patterns closely resembling those of the previously characterized *UGT74AD1*. Furthermore, the co-correlation analyses with crocin levels in different developmental stages highlighted *UGT91P3* and *UGT91K3* as the best candidates, albeit only *UGT91P3* showed higher expression levels in the stigma compared with other tissues, whereas *UGT91K3* was characterized by higher expression levels in leaves, where crocins are not produced, thus suggesting that other metabolites act as substrates for *UGT91K3* [34,35] in saffron vegetative tissues.

The activity of UGT91P3 was tested in vitro using bacteria extracts and in vivo through transient expression in *N. benthamiana* [36]. UGT91P3 was not active against crocetin, but when the activity of UGT91P3 was tested with UGT74AD1 over crocetin, it was possible to detect crocins with more than two glucose molecules, such as crocin-3Gg, crocin-4GG, together with crocin-2G, which was also detected in the reaction. In addition, UGT91P3 was incubated alone with crocin-3Gg, catalyzing the formation of crocin-4GG. The obtained products in the two different reactions suggest that crocin-1g, crocin-2gg and crocin-3Gg are substrates for UGT91P3. The activity of UGT91P3 was also tested in *N. benthamiana* leaves by transient expression, together with additional crocin pathway enzymes. It was previously described that the transient or stable expression of *CsCCD2L* in *N. benthamiana* plants allowed the accumulation of crocins [33,37], which are produced from crocetin by the action of endogenous UGTs. However, the co-expression of the specific UGTs in saffron allowed for greater efficiency in terms of crocin levels, and the production of new ones, which were not detected when *CsCCD2L* was expressed alone. The co-expression of *UGT91P3* was associated with a higher accumulation of crocins with a higher glucosylation degree (more glucose molecules), proving the activity of this UGT in planta.

Previously, other UGTs involved in the sugar chain elongation of crocins have been identified in gardenia [17] and Buddleja [18]; in both cases, the identified UGTs belonged to the UGT94 subfamily, although the identity between these enzymes was below 52%. Interestingly, UGT91P3 showed only 32–33% identity with these enzymes. These findings confirm that the recruitment of enzymes from different subfamilies in the pathway of crocin biosynthesis, such as the UGTs and the CCDs [1,8,17], points out the parallel evolution [38] of this pathway in monocots (saffron) and dicots (Buddleja and gardenia). 

One of the important and most unique properties of UGTs is that they can form homo- and hetero-oligomers [39,40]. Furthermore, it has long been established that successive enzymatic steps are often catalyzed by physically interacting proteins forming permanent or transient multi-enzyme complexes in the cellular environment [41], where intermediates are passed on from one enzyme to the next, referred to as metabolic channeling, leading to an optimized metabolic flux [42]. In addition, it was found that these enzyme complexes are often associated with intra-cellular membrane systems. UGT74AD1 seems to be associated with membrane structures [43], and it was also detected in a proteome of chromoplast from saffron [11], suggesting an interaction with the external membrane of the chromoplast, where it easily accesses the crocetin substrate, synthesized in the chromoplast by CsCCD2L [9]. In this context, two-hybrid (Y2H) assays were carried out to determine the presence of a UGT-based enzymatic complex taking part in the saffron crocin pathway. Notably, this analysis showed the interaction between UGT74AD1 and UGT91P3, thus suggesting the presence of a metabolon in the cellular environment, which could provide catalytic advantages via substrate channeling, preventing the loss of intermediates (Figure 1) by diffusion and by the reduction of the transit time between active sites, thus protecting the chemically labile intermediates, for the action of other enzymes (in this particular case, the presence of hydrolases in the stigma tissue [44]).

In conclusion, the results from the present study reveal a novel enzymatic activity in the specialized metabolic pathways of crocins in saffron, and allowed for completing the identification of candidate enzymes for all the steps in the biosynthesis of these valuable compounds. From a commercial point of view, the identification of UGT91P3 has important implications, since it provides an additional tool for the industrial exploitation of these apocarotenoids, enabling their higher stability, solubility and, potentially, bioavailability.

## 4. Materials and Methods

### 4.1. Plant Materials

Dissected stigmas were collected at different developmental stages, as previously described [45], and plant tissues from saffron cultivated in the Botanical Garden of Castilla-La Mancha (Albacete, Spain) were used throughout the experiments. All tissues were collected and immediately frozen in liquid nitrogen and stored at −80 °C until required. *N. benthamiana* plants used for agroinfiltration experiments were cultured in soil in a growth chamber maintained at the following conditions: temperatures at 26 ± 2 °C in light and 22 ± 2 °C in dark; photoperiod with a 16 h light, 8 h dark cycle; 120 μmol light intensity with LED lamps.

### 4.2. Chemicals

Crocetin and crocins with three and four glucose molecules were purified from the stigmas at anthesis, by using the method previously described [14,46]. The purity of each purified compound was estimated by HPLC-DAD and LC-HESI-HRMS [33]. Uridine 5-diphosphate UDP was purchased in analytical grade from Sigma Aldrich.

### 4.3. Cloning of C. sativus cDNAs

Total RNA was isolated from orange stigmas using Direct-Zol RNA microprep according to manufacturer’s protocols (www.zymoresearch.com, accessed on 1 July 2020). First-strand cDNAs were synthesized from 5 µg of total RNA by reverse transcription (RT) using an 18-base pair oligo dT primer and a first-strand cDNA synthesis kit (GE Healthcare Life Sciences, www.gelifesciences.com, accessed on 1 July 2020) following manufacturer’s instructions. One microliter of the reverse-transcribed RNA was used for PCR with gene-specific primers (Supplemental Table S1) and using the following cycling program: one cycle at 94 °C for 3 min, 35 cycles at 94 °C for 20 s, 58 °C for 20 s, and 72 °C for 2 min, and a final extension at 72 °C for 10 min. The amplified PCR products were analyzed by electrophoresis in agarose and further purified for cloning into pGEM-T (Promega Corporation, Madison, WI, USA). After transformation into competent *E. coli* cells and further plasmid DNA preparation, the clones were sequenced with M13R and M13F primers using an automated DNA sequencer at Macrogen, Spain (Madrid, Spain). Sequence analyses were performed with the BLAST suite of programs of the National Centre for Biotechnology Information (NCBI; http://www.ncbi.nlm.nih.gov, accessed on 1 July 2020). Localization searches were carried out using SignalP (http://www.cbs.dtu.dk/services/SignalP, accessed on 1 July 2020).

### 4.4. Protein Sequence and Phylogenetic Analysis

Protein sequences were all obtained from the GenBank database (http://www.ncbi.nlm.nih.gov/genbank/, accessed on 1 July 2020) via the selection of previously characterized UGTs that catalyze chain elongations. Using MEGA 7.0 (https://www.megasoftware.net/, accessed on 1 July 2020), a phylogenetic tree was constructed using the neighbor-joining method based on the Jones–Taylor–Thornton matrix-based model. Bootstrap support values for the tree topology were calculated from 2500 replicates.

### 4.5. Topology Alignment

Relationship of saffron UGTs with known proteins in the PDB were analyzed through the Phyre server (http://www.sbg.bio.ic.ac.uk/phyre2/, accessed on 1 July 2020). The c6jtdb model (pdb), which showed a 27% identity, was used to construct the protein structures. The three-dimensional structures of proteins were further used to check the structural similarity and conserved regions in saffron UGTs. Structural superimposition was performed using the Chimera tool to find conserved structural folds (https://www.cgl.ucsf.edu/chimera/, accessed on 1 July 2020).

### 4.6. RNA Isolation and RT-qPCR Analysis

Plant tissues were ground into a fine powder under liquid nitrogen, and total RNA extraction and first-strand cDNA synthesis were performed as described in the above section. Amplification reactions were set up at a final volume of 25 µL in GoTaq^®^ qPCR Master Mix (Promega, Madison, WI, USA), according to manufacturer’s instructions and using a specific set of oligonucleotides (Supplemental Table S1). The 18SrRNA and Actin transcripts were used as reference genes [45,47]. The cycling parameters were as follows: initial denaturation at 94 °C for 4 min; 30 cycles of denaturation at 94 °C for 20 s, annealing at 60 °C for 20 s and extension at 72 °C for 20 s; and a final extension at 72 °C for 5 min. Expression levels were calculated as previously described [33]. The experiment was repeated with three biological and two technical replicates for each gene.

### 4.7. HPLC-DAD Analysis of Crocins in Saffron Stigmas

Samples were prepared as previously described [33]. In brief, dissected stigmas were ground in liquid nitrogen with the mixer mill MM400 (Retsch GmbH, Haan, Germany) and extracted with 1 mL Tris-HCl (50 mM, pH 7.5, and containing 1 M NaCl), incubated on ice and centrifuged for clarification. The aqueous phases were recovered and analyzed in triplicate by HPLC analysis, as previously described [27]. The different crocins were identified on the basis of HPLC retention times and UV-visible light spectra [5,48], and their concentrations calculated as previously described [49].

### 4.8. Heterologous Expression of UGT91P3 in E. coli and In Vitro Assays

The full-length open reading frame of UGT91P3 was amplified by PCR using TaKaRa Ex Taq DNA Polymerase (Takara BioEurope) and specific oligonucleotides (Supplemental Table S1). The obtained products were cloned in-frame into the pBAD-Thio vector (Invitrogen) by recombination using the In-Fusion^®^ HD Cloning Plus CE kit (Clontech, www.clontech.com, accessed on 1 July 2020). The integrity of the obtained plasmid, pThioUGT91P3, was confirmed by sequencing. The fusion protein was expressed in *E. coli* BL21 cells transformed with the plasmid pGro7 (Takara Bio Inc.; Mobitec, Göttingen, Germany), which enables the co-expression of the groES–groEL–chaperone system. Bacterial growth, protein induction and the preparation of soluble supernatants were carried out as previously described [50]. To study the enzymatic activity of UGT91P3, the soluble supernatants of overexpressing *E. coli* cells (50 µL) were incubated as follows: all the assays were carried out in 200 μL of 100 mM HEPES pH 7.8 containing 1 mM TCEP, 2.5 mM UDP-glucose, and the corresponding substrates (40 μM). The glucosyltransferase activity assays were carried out at 30 °C for 60 min. Subsequently, an equal volume of methanol was added to the samples and centrifuged at 12,000× *g* for 10 min to collect the supernatant, which was analyzed by reverse-phase HPLC as previously described [27]. The reaction products were also analyzed by HPLC-MS, as above described.

### 4.9. Transient Expression in N. benthamiana Leaves

The pDGB3Ω1 ((PNos: Hyg: TNos): (P35S:CsCCD2L:T35S)), and the pDGB3Ω1 ((pNos:Hyg:TNos):(PAtUbQ10:UGT74AD1:T35S) and (pNos:Hyg:TNos):( pAtUBQ3:UGT91P3:T35S)), were constructed following the GB4.0 assembly strategy [51]. Briefly, the first step was the domestication of *UGT91P3* and *UGT74AD1* by removing the internal BsaI and BsmBI sites and adding the adapters. *CsCCD2L* was previously domesticated [9,33]. PCR amplifications of *UGT91P3* and *UGT74AD1*, using GB-adapted primers designed by GB4.0 tools (https://gbcloning.upv.es, accessed on 1 July 2020), were performed using pGEMT-UGT91P3 and pGEMT-UGT74AD1 as templates. The resulting PCR fragments were cloned into the pUPD2 vector to yield domesticated GB parts using a BsmBI restriction–ligation reaction as previously described [9]. The assembly of the domesticated GB parts in the destination plasmid was performed as described [51]. Several assembly construct combinations were performed via restriction–ligation reactions. *E. coli* was transformed with the resulting constructs, and positive white clones were selected under kanamycin (50 µg/mL) for the pDGB3α1/2 constructs or spectinomycin (50 µg/mL) for the pDGB3Ω1/2 constructs, then further confirmed by digestion and sequencing using an automated DNA sequencer (ABI PRISM 3730xl, Perkin Elmer, Macrogen Inc., Seoul, Korea). *Agrobacterium tumefaciens* strain GV3101 was electroporated with the constructs and selected on YEB agar with the corresponding antibiotics. Transient expression experiments were carried out in Nicotiana benthamiana leaves as previously described [33]. As controls, transformations of leaves with single constructs and with the empty vector were performed. Eight-day-agroinfiltrated leaves were lyophilized prior to LC-DAD-HESI-HRMS, and lyophilized samples were used for the analyses of polar metabolites as previously described, then extracted in cold 50% MeOH.

### 4.10. LC-DAD-HESI-HRMS Analysis of Crocins in UGT Assays

UGT assay samples were analyzed using high-performance liquid chromatography–diode array detector–high-resolution mass spectrometry (LC-DAD-HESI-HRMS) as previously described [12,33]. Metabolites were identified by co-migration with standards, and matching the UV spectrum of each peak against those of the standards (on the basis of data from the literature) and *m*/*z* accurate masses, as reported in the PubChem database (http://pubchem.ncbi.nlm.nih.gov/, accessed on 1 July 2020) for monoisotopic mass identification, and/or using the Metabolomics Fiehn Lab Mass Spectrometry Adduct Calculator (http://fiehnlab.ucdavis.edu/staff/kind/Metabolomics/MS-Adduct-Calculator/, accessed on 1 July 2020) in the case of adduct detection. The apocarotenoids were quantified by integrating the peak areas that were converted to concentrations by comparison with the standards and as reported previously [37].

### 4.11. Yeast Two-Hybrid (Y2H) Analysis

Yeast two-hybrid (Y2H) analysis was carried out according to the protocol of the MATCHMAKER Gold (Takara). DNA binding domain (BD) fusion vector pGBKT7 and activation domain (AD) fusion vector pGADT7 have been designed for the identification and confirmation of protein interactions. Bait and prey proteins can be expressed as GAL4 fusions with c-Myc and hemagglutinin epitope tags in these vectors, respectively. We fused UGT91P3 with the DNA BD of pGBKT7 and UGT74AD1 with the DNA AD of pGADT7, using the primer pairs listed in Supplemental Table S1 and infusion reactions (Takara). The UGT91P3+pGBKT7 and UGT74AD1+pGADT7 constructs were transformed by electroporation in Y2HGold and Y187 yeast strains, respectively. The transformed positive cells carrying UGT91P3+pGBKT7 and UGT74AD1+pGADT7 were mated, and the obtained diploid cells were grown on selective medium lacking leucine and tryptophan and supplemented with Aureobasidin A and X-α-Gal to score protein–protein interactions. A genuine interaction test was performed with yeast cells spotted on selective medium lacking tryptophan, histidine and leucine, supplemented with Aureobasidin A in a series of 1:10 dilutions starting with an OD600 of 2.

### 4.12. Data Integration

Co-expression and correlation network analyses were performed as previously described [11,12].

## Figures and Tables

**Table 1:** List of saffron apocarotenoids found in the stigma and in the in vitro reactions in the HPLC-DAD chromatograms, detected at 440 nm, separated with a C18 column, and identified by high-resolution mass spectrometry (HRMS) analyses. *RT*: retention time. *M*/*Z*: detected mass*/*charge.

*Name*	*RT*	*M/Z*
***t-4GG***	11.17	977.38590 [M + H], 994.41250 [M + NH_4_]
***t-3Gg***	12.05	815.33310 [M + H], 832.35970 [M + NH_4_]
***t-2gg***	13.14	653.28030 [M + H], 670.30690 [M + NH_4_]
***c-5nG***	13.91	1156.46530 [M + H]
***c-4GG***	14.50	977.38590 [M + H], 994.41250 [M + NH_4_]
***t-2gg***	15.05	653.28030 [M + H], 670.30690 [M + NH_4_]
***c-3Gg***	15.55	815.33310 [M + H], 832.35970 [M + NH_4_]
***t-1g***	16.75	491.22750 [M + H], 508.25410 [M + NH_4_]
***t-1g***	20.48	491.22750 [M + H], 508.25410 [M + NH_4_]
***t-crocetin***	19.93	329.17470 [M + H]
***c-crocetin***	22.49	329.17470 [M + H]

## Data Availability

Immediately after publication.

## Supplementary Materials

Supplementary materials can be found at https://www.mdpi.com/article/10.3390/ijms22168815/s1.

## References

[1] Ahrazem O., Diretto G., Argandona J., Rubio-Moraga A., Julve J.M., Orzaez D., Granell A., Gomez-Gomez L. (2017). Evolutionarily distinct carotenoid cleavage dioxygenases are responsible for crocetin production in Buddleja davidii. J. Exp. Bot..

[2] Ahrazem O., Rubio-Moraga A., Nebauer S.G., Molina R.V., Gomez-Gomez L. (2015). Saffron: Its Phytochemistry, Developmental Processes, and Biotechnological Prospects. J. Agric. Food Chem..

[3] Christodoulou E., Kadoglou N.P., Kostomitsopoulos N., Valsami G. (2015). Saffron: A natural product with potential pharmaceutical applications. J. Pharm. Pharmacol..

[4] Moraga A.R., Nohales P.F., Perez J.A., Gomez-Gomez L. (2004). Glucosylation of the saffron apocarotenoid crocetin by a glucosyltransferase isolated from *Crocus sativus* stigmas. Planta.

[5] Rubio-Moraga A., Trapero A., Ahrazem O., Gomez-Gomez L. (2010). Crocins transport in *Crocus sativus*: The long road from a senescent stigma to a newborn corm. Phytochemistry.

[6] Ahrazem O., Diretto G., Argandona Picazo J., Fiore A., Rubio-Moraga A., Rial C., Varela R.M., Macias F.A., Castillo R., Romano E. (2019). The Specialized Roles in Carotenogenesis and Apocarotenogenesis of the Phytoene Synthase Gene Family in Saffron. Front. Plant Sci..

[7] Ahrazem O., Gomez-Gomez L., Rodrigo M.J., Avalos J., Limon M.C. (2016). Carotenoid Cleavage Oxygenases from Microbes and Photosynthetic Organisms: Features and Functions. Int. J. Mol. Sci..

[8] Frusciante S., Diretto G., Bruno M., Ferrante P., Pietrella M., Prado-Cabrero A., Rubio-Moraga A., Beyer P., Gomez-Gomez L., Al-Babili S. (2014). Novel carotenoid cleavage dioxygenase catalyzes the first dedicated step in saffron crocin biosynthesis. Proc. Natl. Acad. Sci. USA.

[9] Ahrazem O., Rubio-Moraga A., Berman J., Capell T., Christou P., Zhu C., Gomez-Gomez L. (2016). The carotenoid cleavage dioxygenase CCD2 catalysing the synthesis of crocetin in spring crocuses and saffron is a plastidial enzyme. New Phytol..

[10] Gomez-Gomez L., Pacios L.F., Diaz-Perales A., Garrido-Arandia M., Argandona J., Rubio-Moraga A., Ahrazem O. (2018). Expression and Interaction Analysis among Saffron ALDHs and Crocetin Dialdehyde. Int. J. Mol. Sci..

[11] Gomez-Gomez L., Parra-Vega V., Rivas-Sendra A., Segui-Simarro J.M., Molina R.V., Pallotti C., Rubio-Moraga A., Diretto G., Prieto A., Ahrazem O. (2017). Unraveling Massive Crocins Transport and Accumulation through Proteome and Microscopy Tools during the Development of Saffron Stigma. Int. J. Mol. Sci..

[12] Ahrazem O., Argandona J., Fiore A., Aguado C., Lujan R., Rubio-Moraga A., Marro M., Araujo-Andrade C., Loza-Alvarez P., Diretto G. (2018). Transcriptome analysis in tissue sectors with contrasting crocins accumulation provides novel insights into apocarotenoid biosynthesis and regulation during chromoplast biogenesis. Sci. Rep..

[13] Dufresne C., Cormier F., Dorion S. (1997). In vitro formation of crocetin glucosyl esters by *Crocus sativus* callus extract. Planta Med..

[14] Ahrazem O., Rubio-Moraga A., Jimeno M.L., Gomez-Gomez L. (2015). Structural characterization of highly glucosylated crocins and regulation of their biosynthesis during flower development in Crocus. Front. Plant Sci..

[15] Pfister S., Steck A., Pfander H. (1996). Isolation and structure elucidation of carotenoid glycoslyesters on gardenia fruits (Gardenia jasminoides) and saffron (*Crocus sativus*). J. Agric. Food Chem..

[16] Nagatoshi M., Terasaka K., Owaki M., Sota M., Inukai T., Nagatsu A., Mizukami H. (2012). UGT75L6 and UGT94E5 mediate sequential glucosylation of crocetin to crocin in Gardenia jasminoides. FEBS Lett..

[17] Xu Z., Pu X., Gao R., Demurtas O.C., Fleck S.J., Richter M., He C., Ji A., Sun W., Kong J. (2020). Tandem gene duplications drive divergent evolution of caffeine and crocin biosynthetic pathways in plants. BMC Biol..

[18] Diretto G., López-Jiménez A.J., Ahrazem O., Frusciante S., Song J., Rubio-Moraga Á., Gómez-Gómez L. (2021). Identification and characterization of apocarotenoid modifiers and carotenogenic enzymes for biosynthesis of crocins in Buddleja davidii flowers. J. Exp. Bot..

[19] De Bruyn F., Maertens J., Beauprez J., Soetaert W., De Mey M. (2015). Biotechnological advances in UDP-sugar based glycosylation of small molecules. Biotechnol. Adv..

[20] Bowles D., Lim E.K., Poppenberger B., Vaistij F.E. (2006). Glycosyltransferases of lipophilic small molecules. Annu. Rev. Plant Biol..

[21] Tiwari P., Sangwan R.S., Sangwan N.S. (2016). Plant secondary metabolism linked glycosyltransferases: An update on expanding knowledge and scopes. Biotechnol. Adv..

[22] Lim E.-K., Bowles D.J. (2004). A class of plant glycosyltransferases involved in cellular homeostasis. EMBO J..

[23] Osmani S.A., Bak S., Moller B.L. (2009). Substrate specificity of plant UDP-dependent glycosyltransferases predicted from crystal structures and homology modeling. Phytochemistry.

[24] Song C., Gu L., Liu J., Zhao S., Hong X., Schulenburg K., Schwab W. (2015). Functional Characterization and Substrate Promiscuity of UGT71 Glycosyltransferases from Strawberry (Fragaria × ananassa). Plant Cell Physiol..

[25] Meech R., Hu D.G., McKinnon R.A., Mubarokah S.N., Haines A.Z., Nair P.C., Rowland A., Mackenzie P.I. (2019). The UDP-Glycosyltransferase (UGT) Superfamily: New Members, New Functions, and Novel Paradigms. Physiol. Rev..

[26] Huang F.-C., Giri A., Daniilidis M., Sun G., Härtl K., Hoffmann T., Schwab W. (2018). Structural and Functional Analysis of UGT92G6 Suggests an Evolutionary Link Between Mono- and Disaccharide Glycoside-Forming Transferases. Plant Cell Physiol..

[27] Moraga A.R., Rambla J.L., Ahrazem O., Granell A., Gomez-Gomez L. (2009). Metabolite and target transcript analyses during *Crocus sativus* stigma development. Phytochemistry.

[28] Tripathi S., Jadaun J.S., Chandra M., Sangwan N.S. (2016). Medicinal plant transcriptomes: The new gateways for accelerated understanding of plant secondary metabolism. Plant Genet. Resour..

[29] Ahrazem O., Rubio-Moraga A., Argandona-Picazo J., Castillo R., Gomez-Gomez L. (2016). Intron retention and rhythmic diel pattern regulation of carotenoid cleavage dioxygenase 2 during crocetin biosynthesis in saffron. Plant Mol. Biol..

[30] Ahrazem O., Argandona J., Fiore A., Rujas A., Rubio-Moraga A., Castillo R., Gomez-Gomez L. (2019). Multi-species transcriptome analyses for the regulation of crocins biosynthesis in Crocus. BMC Genom..

[31] Bourne Y., Henrissat B. (2001). Glycoside hydrolases and glycosyltransferases: Families and functional modules. Curr. Opin. Struct. Biol..

[32] Itkin M., Davidovich-Rikanati R., Cohen S., Portnoy V., Doron-Faigenboim A., Oren E., Freilich S., Tzuri G., Baranes N., Shen S. (2016). The biosynthetic pathway of the nonsugar, high-intensity sweetener mogroside V from Siraitia grosvenorii. Proc. Natl. Acad. Sci. USA.

[33] Diretto G., Ahrazem O., Rubio-Moraga A., Fiore A., Sevi F., Argandona J., Gomez-Gomez L. (2019). UGT709G1: A novel uridine diphosphate glycosyltransferase involved in the biosynthesis of picrocrocin, the precursor of safranal in saffron (*Crocus sativus*). New Phytol..

[34] Williams C.A., Harborne J.B., Goldblatt P. (1986). Correlations between phenolic patterns and tribal classification in the family iridaceae. Phytochemistry.

[35] Mykhailenko O., Ivanauskas L., Bezruk I., Sidorenko L., Lesyk R., Georgiyants V. (2021). Characterization of Phytochemical Components of *Crocus sativus* Leaves: A New Attractive By-Product. Sci. Pharm..

[36] Petit E., Berger M., Camborde L., Vallejo V., Daydé J., Jacques A. (2020). Development of screening methods for functional characterization of UGTs from Stevia rebaudiana. Sci. Rep..

[37] Martí M., Diretto G., Aragonés V., Frusciante S., Ahrazem O., Gómez-Gómez L., Daròs J.-A. (2020). Efficient production of saffron crocins and picrocrocin in Nicotiana benthamiana using a virus-driven system. Metab. Eng..

[38] Weng J.-K. (2014). The evolutionary paths towards complexity: A metabolic perspective. New Phytol..

[39] Fujiwara R., Yokoi T., Nakajima M. (2016). Structure and Protein-Protein Interactions of Human UDP-Glucuronosyltransferases. Front. Pharmacol..

[40] Wilson A.E., Tian L. (2019). Phylogenomic analysis of UDP-dependent glycosyltransferases provides insights into the evolutionary landscape of glycosylation in plant metabolism. Plant J..

[41] Durek P., Walther D. (2008). The integrated analysis of metabolic and protein interaction networks reveals novel molecular organizing principles. BMC Syst. Biol..

[42] Ovádi J., Srere P.A. (2000). Macromolecular compartmentation and channeling. Int. Rev. Cytol..

[43] Demurtas O.C., Frusciante S., Ferrante P., Diretto G., Azad N.H., Pietrella M., Aprea G., Taddei A.R., Romano E., Mi J. (2018). Candidate Enzymes for Saffron Crocin Biosynthesis Are Localized in Multiple Cellular Compartments. Plant Physiol..

[44] Baba S.A., Mohiuddin T., Basu S., Swarnkar M.K., Malik A.H., Wani Z.A., Abbas N., Singh A.K., Ashraf N. (2015). Comprehensive transcriptome analysis of *Crocus sativus* for discovery and expression of genes involved in apocarotenoid biosynthesis. BMC Genom..

[45] Rubio A., Rambla J.L., Santaella M., Gomez M.D., Orzaez D., Granell A., Gomez-Gomez L. (2008). Cytosolic and plastoglobule-targeted carotenoid dioxygenases from *Crocus sativus* are both involved in beta-ionone release. J. Biol. Chem..

[46] Rubio Moraga A., Ahrazem O., Rambla J.L., Granell A., Gomez Gomez L. (2013). Crocins with high levels of sugar conjugation contribute to the yellow colours of early-spring flowering crocus tepals. PLoS ONE.

[47] Hu J., Liu Y., Tang X., Rao H., Ren C., Chen J., Wu Q., Jiang Y., Geng F., Pei J. (2020). Transcriptome profiling of the flowering transition in saffron (*Crocus sativus* L.). Sci. Rep..

[48] Carmona M., Zalacain A., Sanchez A.M., Novella J.L., Alonso G.L. (2006). Crocetin esters, picrocrocin and its related compounds present in *Crocus sativus* stigmas and Gardenia jasminoides fruits. Tentative identification of seven new compounds by LC-ESI-MS. J. Agric. Food Chem..

[49] Himeno H., Sano K. (1987). Synthesis of crocin, picrocrocin and safranal by saffron stigma-like structures proliferated in vitro. Agric. Biol. Chem..

[50] Alder A., Holdermann I., Beyer P., Al-Babili S. (2008). Carotenoid oxygenases involved in plant branching catalyse a highly specific conserved apocarotenoid cleavage reaction. Biochem. J..

[51] Sarrion-Perdigones A., Vazquez-Vilar M., Palaci J., Castelijns B., Forment J., Ziarsolo P., Blanca J., Granell A., Orzaez D. (2013). GoldenBraid 2.0: A comprehensive DNA assembly framework for plant synthetic biology. Plant Physiol..

