# Supplementary material for: A New Glycosyltransferase Enzyme from Family 91, UGT91P3, Is Responsible for the Final Glucosylation Step of Crocins in Saffron (Crocus sativus L.)
Source: Int J Mol Sci. 2021 Aug 16;22(16):8815. doi: 10.3390/ijms22168815 (PMC8396231; doi:10.3390/ijms22168815)
Supplement: Supplementary file 1 [file ijms-22-08815-s001.zip › ijms-1309546-supplementary.pdf]

Supplemental Figure S1

A

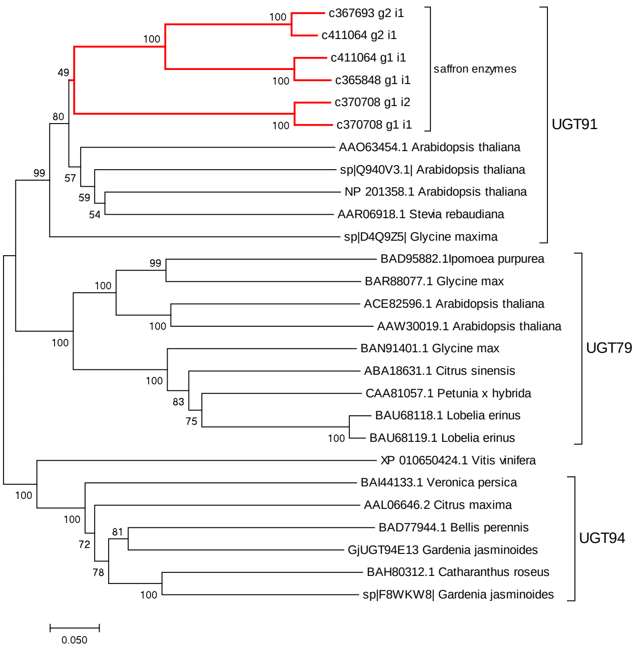

B

Percent Identity Matrix - created by Clustal2.1

c370708_g1_i2	**100.00**	90.46	46.74	46.79	47.87	48.65
c370708_g1_i1	90.46	**100.00**	45.09	45.26	46.67	47.22
c411064_g1_i1	46.74	45.09	**100.00**	96.92	63.46	68.49
c365848_g1_i1	46.79	45.26	96.92	**100.00**	62.50	62.76
c367693_g2_i1	47.87	46.67	63.46	62.50	**100.00**	93.06
c411064_g2_i1	48.65	47.22	68.49	62.76	93.06	**100.00**

C

PSPG BOX

```
c370708_g1_i2   WIPQTKFLAHSSVGGFLTHCGSSSIVEGLSFGLVMVALPMVFTQ
c370708_g1_i1   WIPQTKFLAHSSVGGFLTHCGSSSIVEGLSFGLVMVALPMVFTQ
c411064_g1_i1   WVPQLEVLAHSAVGGFLTHCGLGSIIEGLHFGRPLILMPVRGDQ
c365848_g1_i1   WVPQLEVLAHSAVGGFLTHCGLGSIIEGLHFGRPLILMPVRGDQ
c367693_g2_i1   WVPQMRVLADGSVGGFVTHCGWSSVVESLHFGHPLVLLPIFGDQ
c411064_g2_i1   WVPQMRVLADGSVGGFVTHCGWSSVVESLHFGHPLVLLPIFGDQ
```

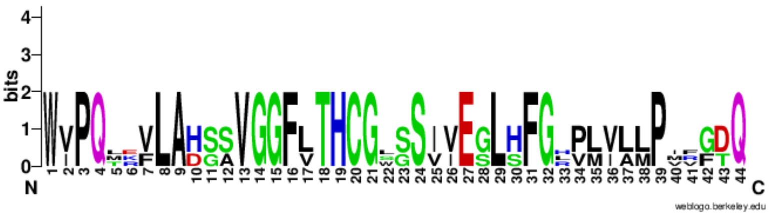

Supplemental Figure S1. Characteristics of the amino acid sequences of the isolated UGTs in saffron stigmas. A) Phylogenetic tree with UGT enzymes from subfamilies UGT79, 91 and 94. B) Percentage identity matrix among the identified UGTs in saffron. C) Consensus PSPG box of the identified UGTs. WebLogo (http://weblogo.berkeley.edu/logo.cgi). Letter size is proportional to the degree of amino acid conservation.

Supplemental Figure S2

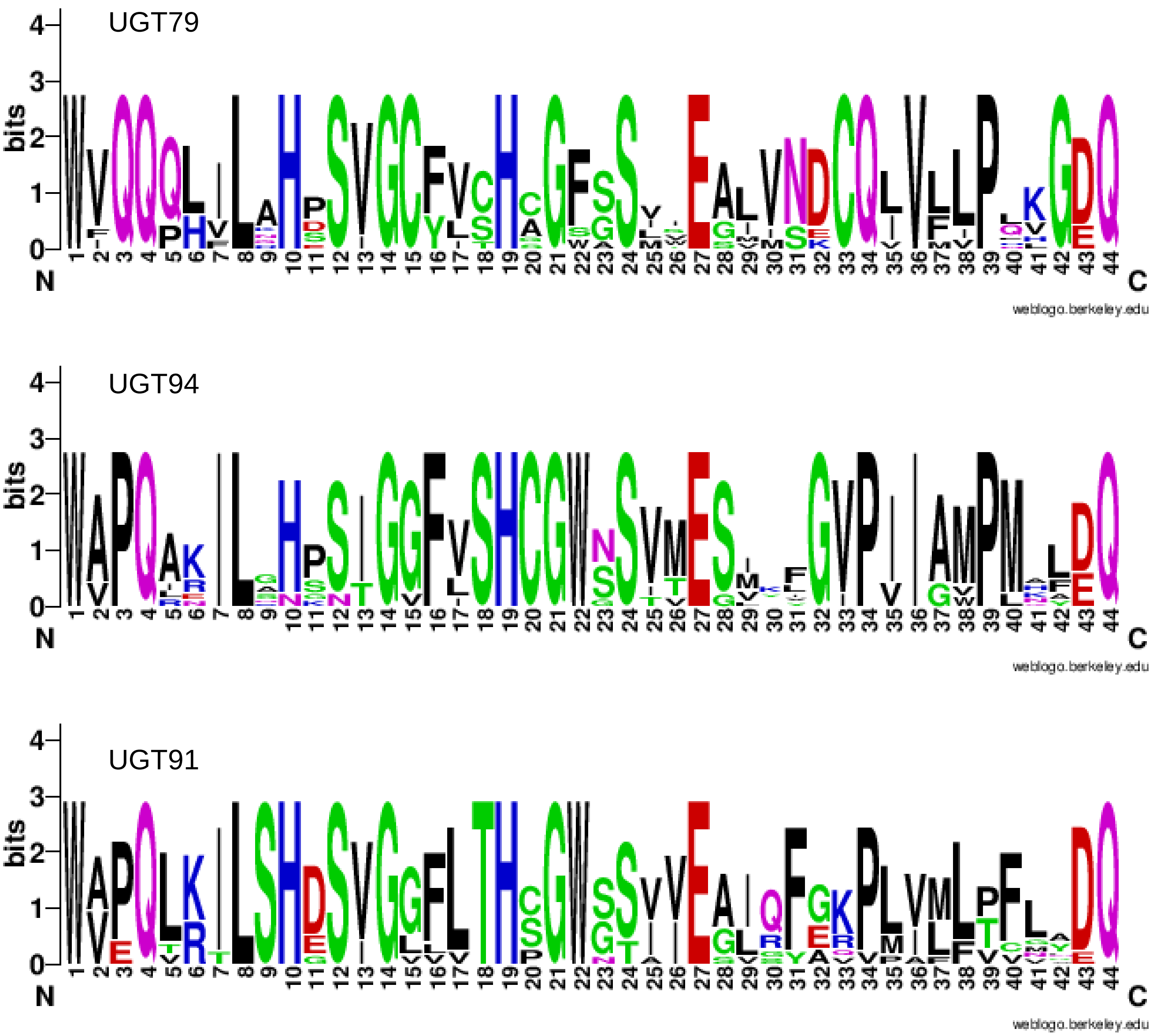

Supplemental Figure S2. WebLogo (http://weblogo.berkeley.edu/logo.cgi) representing the PSPG-box motif of selected UGTs subfamilies. The key amino acid residues interacting directly with the sugar donor are in positions: 1, 2, 3,19 ,22 ,23 ,24 ,27,43 and 44. The amino acid in position 44 is considered to be related to the donor sugar preference of the enzyme, with Q for glucose preference. Letter size is proportional to the degree of amino acid conservation.

Supplemental Figure S3

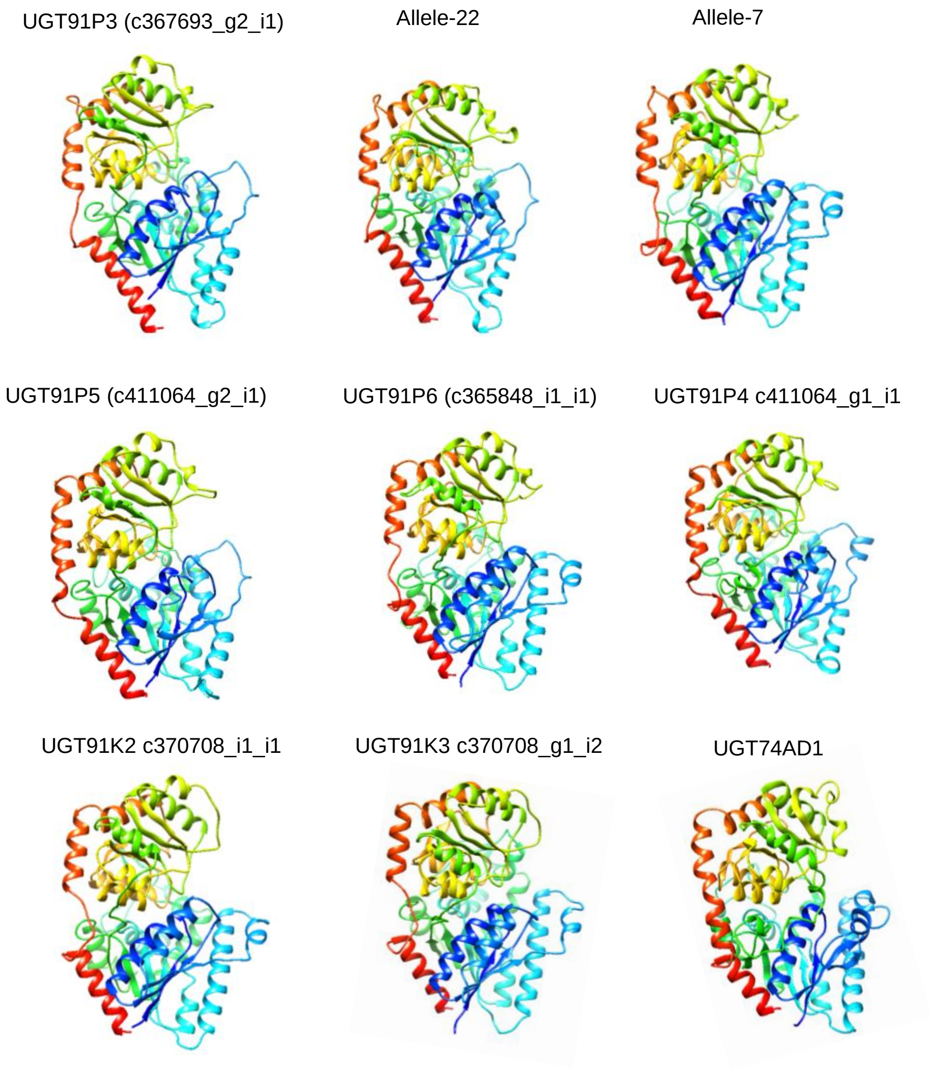

Supplemental Figure S3. 3D protein structure (ribbon diagram) of saffron UGTs. Both backbone helices are in orange and red.

Supplemental Figure S4

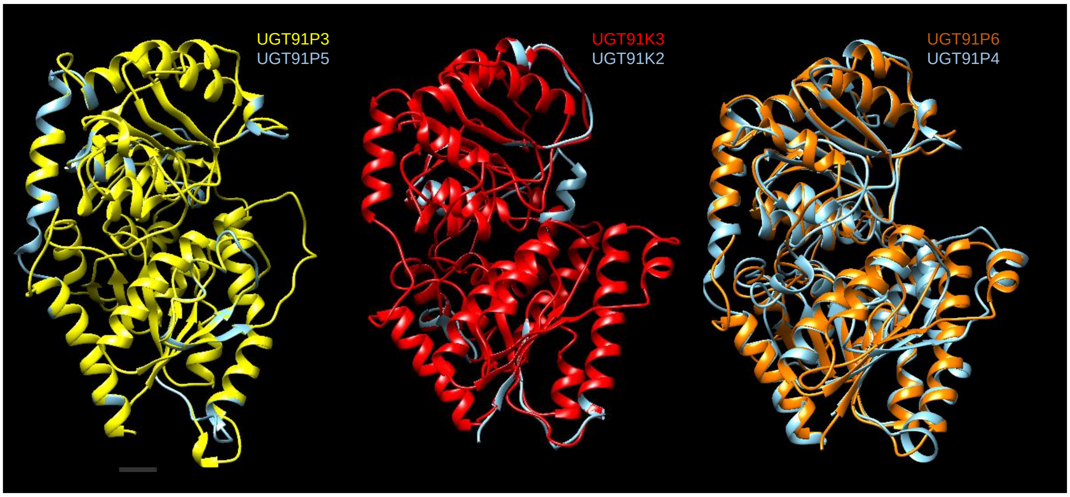

Supplemental Figure S4. Structural optimized superposition of the two model structures of UGTs showing the best geometries. Models obtained by using Chimera (https://www.cgl.ucsf.edu/chimerax/).

Supplemental Figure S5

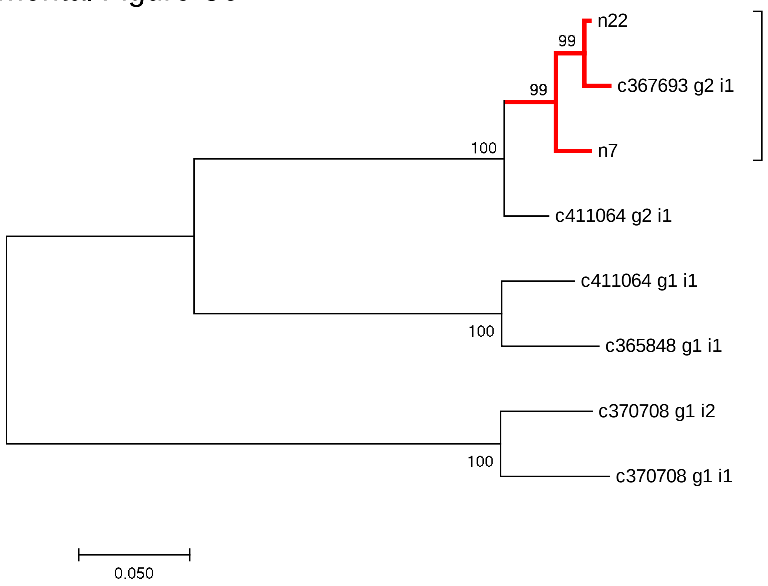

Supplemental Figure S5. Phylogenetic tree of the isolated UGT91P3 alleles based on amino acid sequences.

Supplemental Figure S6

A

```
C367693_g2_i1      MEAGGDKLHIVVFPWLAFGHMLPFLELSKSLAKRGHLISFVSTPKNIQRFPNLPPQISPL 60
7                  MEAGGDKLHIVVFPWLAFGHMLPFLELSKSLAKRGHLISFVSTPKNIQRFPNLPPQISPL 60
22                 MEAGGDKLHIVVFPWLAFGHMLPFLELSKSLAKRGHLISFVSTPKNIQRFPNLPPQISPL 60
                   ************************************************************

C367693_g2_i1      INFIPLSLPKVEGMPGDVEATTDLSPANLQYLKKALDGLEQPFRSFLREASPKPDWIIQD 120
7                  INFIPLSLPKVEGMPGDVEATTDLPPANLQYLKKALDGLEQPFRSFLREASPKPDWIIQD 120
22                 INFIPLSLPKVEGMPGDVEATTDLPPANLQYLKKALDGLEQPFRSFLREASPKPDWIIQD 120
                   ************************ ***********************************

C367693_g2_i1      LLQHWIPPIAAELHVPSMYFGTVPAAALTFFGHPSQLSSRGKGLEGWLASPPWVPFPSKV 180
7                  LLQHWIPPIAAELHVPSMYFGTVPAAALTFFGHPSEFSKRKKGIEDWLVSPPWVPFPSKV 180
22                 LLQHWIPPIAAELHVPSMYFGTVPAAALTFFGHPSEFSKRSKGIEGWLGSPPWVPFPSKV 180
                   ***********************************::*.* **:*.** ***********

C367693_g2_i1      AYRLHELIVMAKDAAGPLHSGMTDARRMEAAIVGCCAVAIRTCRELESEWLPILEEIYGK 240
7                  AYRLHEMIVMAKDTAGPLHSGVTDVRRMEAAIVGCCAVAIRTCRELESEWLPILEEIYGK 240
22                 AYRLHELIVMAKDAAGPLHSGVTDARRMEAAIVGCCAVAIRTCRELESEWLPILEEIYGK 240
                   ******:******:*******:**.***********************************

C367693_g2_i1      PVIPVGLLLPTADESTDGNSIIDWLGTRSQESVVYIALGSEVSIGVELIHELALGLELAG 300
7                  PVIPVGLLLPTADESTDGNSIIDWLGTRSQESVVYIALGSEVSIGVELIHELALGLELAG 300
22                 PVIPVGLLLPTADESTDGNSIIDWLGTRSQESVVYIALGSEVSIGVELIHELALGLELAG 300
                   ************************************************************

C367693_g2_i1      LPFLWALRRPYGLSSDTEILPGGFEERTRGYGKVVMGWVPQMRVLADGSVGGFVTHCGWS 360
7                  LPFLWALRRPYGLSSDTEILPGGFEERTRGYGKVVMGWVPQMRVLADRSVGGFLTHCGWS 360
22                 LPFLWALRRPYGLSSDTEILPGGFEERTRGYGKVVMGWVPQMRVLADRSVGGFVTHCGWS 360
                   *********************************************** *****:******

C367693_g2_i1      SVVESLHFGHPLVLLPIFGDQGLNARLLEEKGIGVEVERKGDASFTRNEVAKAVNLVMVE 420
7                  SVVESLHFGHPLVLLPIFGDQGLNARLLEEKGIGVEVERKGDGSFTRNEVAKAINLIMVE 420
22                 SVVESLHFGHPLVLLPIFGDQGLNARLLEEKGIGVEVERKGDGSFTRNEVAKAINLIMVE 420
                   ******************************************.**********:**:***

C367693_g2_i1      GDGSGSSYRKKAKEMKKIFGDKECQEKYVDEFIQFLLSNGTAKG 464
7                  GDGSGSSYRKKAKEMKKIFADKECQEKYVDEFVQFLLSNGTAKG 464
22                 GDGSGSSYRKKAKEMKKIFADKECQEKYVDEFVQFLLSNGTAKG 464
                   *******************.************:***********
```

B

```
Percent Identity Matrix - created by Clustal2.1
1: n°7            100.00  96.98   95.91
2: n°22            96.98 100.00   98.49
3: c367693_g2_i1   95.91  98.49  100.00
```

Supplemental Figure S6. Multiple sequence alignment of UGt91P3 alleles and percentage identity among the UGt91P3 alleles. A) Sequence alignment generated with EBI Clustal Omega tool (https://www.ebi.ac.uk/Tools/msa/clustalo/). Black box marks major differences among the three sequences, these amino acids are localized in a loop region, which participates in the formation of the sugar acceptor pocket. B) Percentage identity matrix for the three alleles.

Supplemental Figure S7

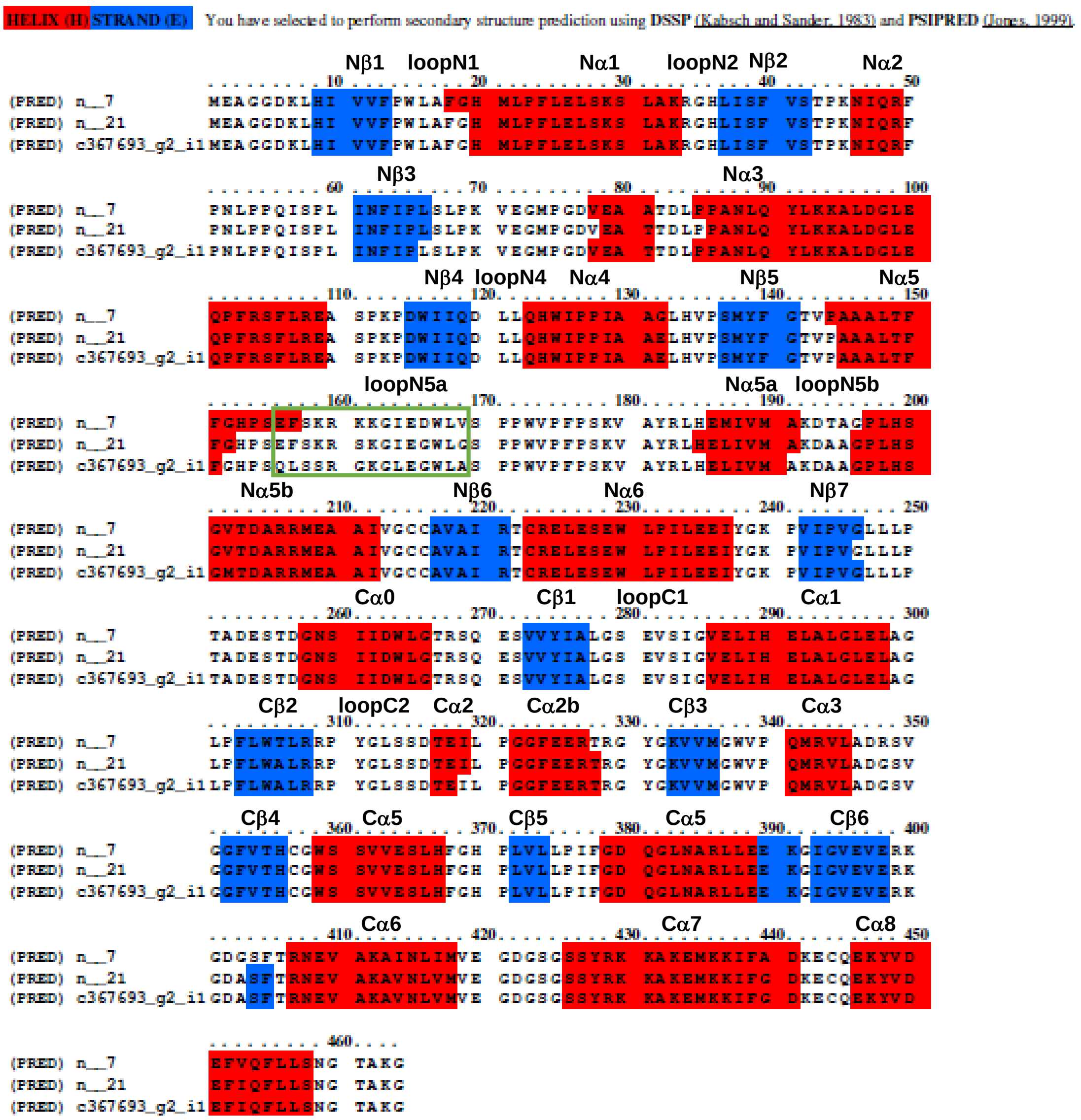

Supplemental Figure S7. Structure-based sequence alignment of UGT91P3 alleles. The secondary structure elements are shown above the alignment as α-helices (red), β-sheets (blue). Green box marks major differences among the three alleles.

Supplemental Figure S8

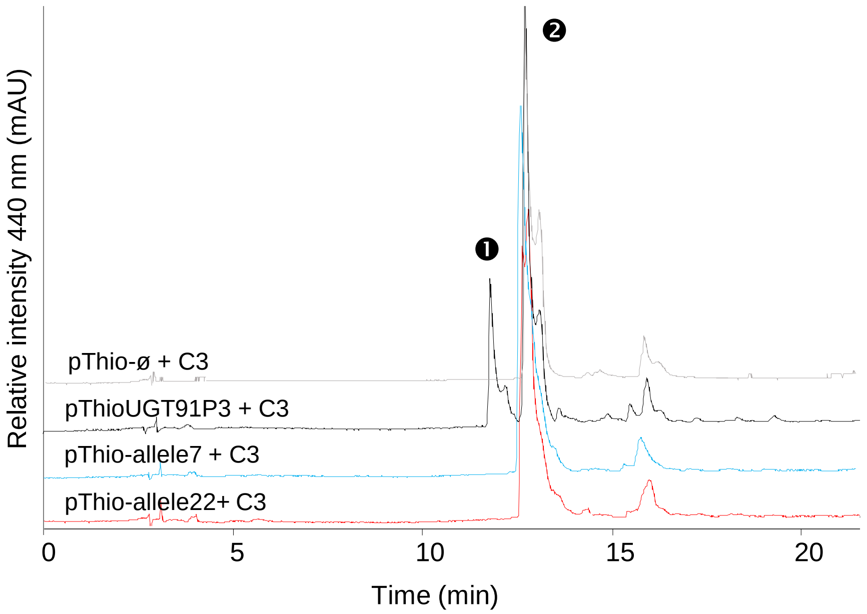

Supplemental Figure S8. Enzyme Activity of UGT91P3 alleles. The UGTs were expressed in *E. coli* and protein extracts were assayed for activity with crocin t-3Gg as substrate. As controls, the assay was performed with protein extracts of *E. coli* transformed with the empty vector. Products were then analysed using HPLC-DAD and by HESI-HRMS (for more detail, see materials and methods). UV detection was monitored at 440 nm.

Supplemental Table S1. Oligonucleotide sequences using in this study.

	Forward 5′ to 3′	Reverse 5′ to 3′
**UGT91P3**		
Cloning	ATGGAAGCTGGTGGTGATAAACT	CTACCCTTTTGCTGTTCCATTAC
pTHIO-cloning.	cgcccttggcgaattc ATGGAAGCTGGTGGTGATAAACT	taccctcgaggaattcCTACCCTTTTGCTGTTCCATTAC
Expression	CAGTTGTCGAGCCGCGGT	CCTTTTGCTGTTCCATTACT
GBUGT91P3	gcgccgtctcgctcgaATGGAAGCTGGTGATGACAAG	gcgccgtctcgctcaaagcTTATAGTGAGGTGGTTCTGTG
Two hybrid	catggaggccgaattcATGGAAGCTGGTGGTGATAAAC	gcaggtcgacggatccCTACCCTTTGCTGTTCCATTAC
**UGT74AD1**		
Two hybrid	gagtggccattatggcccATGTTGAACGGCAACAAATGC	ccgaggcggccgacatgTTAAACTAAGGAAATTTTGGAGT
Expression	ACCTAGTCGTCGGGCCTATT	GGCGGTAGATTGTGTCCACT
GBUGT74AD1	gcgccgtctcgctcgaATGTTGAACGGCAACAAATGC	gcgccgtctcgctcaaagcTTAAACTAAGGAAATTTTGGAGTCAT
**Expression**		
Expression UGT91P5	CGAAGGCGGTGGAGTTAGT	TTATGAGGTGGTTGTTCTGT
Expression UGT91P4	AGGCTAAAGGATTTGCAAAC	GTAGCGGCGAAGCTGATCAT
Expression UGT91P6	GGAGATGAAGACGGTGCTCG	GACTCCACAGGTGTTGTATC
Expression UGT91K2	GAGATGTCGGCAGAGGGAT	ACTCTTACCACTTGCCGCTT
Expression UGT91K3	GAGTGATCTCCCAAGAGGC	GATCATGTCACTTTGAACTT
